# Association Between Parental eHealth Literacy and Self-Reported Child Heat-Protection Practices: A Cross-Sectional Study in Türkiye

**DOI:** 10.3390/healthcare14183038

**Published:** 2026-09-16

**Authors:** İsa Çelik, Fatma Dinç, Mustafa Belli, Ayten Yılmaz Yavuz, Murat Bektaş

**Affiliations:** 1 Department of Nursing, Faculty of Health Sciences, Recep Tayyip Erdoğan University, Rize 53100, Türkiye; ayten.yilmaz@erdogan.edu.tr; 2 Department of Nursing, Faculty of Health Sciences, Bartın University, Bartın 74100, Türkiye; fatmadinc@bartin.edu.tr; 3 Department of Pediatric Nursing, Faculty of Health Sciences, Burdur Mehmet Akif Ersoy University, Burdur 15200, Türkiye; mbelli@mehmetakif.edu.tr; 4 Faculty of Nursing, Dokuz Eylül University, İzmir 35340, Türkiye; murat.bektas@deu.edu.tr

**Keywords:** climate change, extreme heat, child health, parents, eHealth literacy, protective practices, public health

## Abstract

**Background/Objectives:** Extreme heat events pose substantial health risks to children, who depend on their caregivers for protection. Although parents increasingly use digital health information, evidence on the relationship between parental eHealth literacy and heat-protection practices remains limited. This study examined the association between parental eHealth literacy and self-reported practices for protecting children from extreme heat. **Methods:** A cross-sectional online survey was conducted with 313 parents in Türkiye between August and September 2023 using convenience and snowball sampling. Data were collected using the Turkish eHealth Literacy Scale and a researcher-developed 33-item heat-protection practices survey. Descriptive statistics, exploratory item-level and dimensionality analyses, developmental sensitivity analyses, and regression analyses were performed. **Results:** Participants were predominantly female (85.3%) and university graduates (90.4%). The mean eHealth literacy score was 33.15 ± 5.25, and the mean heat-protection practice score was 137.74 ± 14.92. In this sample, 25.6% of parents reported that their children had become ill more frequently during the summer due to hot weather, and 49.8% reported increased heat-related household expenses. Higher parental eHealth literacy was associated with higher self-reported heat-protection practice scores (*B* = 0.993, *SE* = 0.151, β = 0.350, 95% CI: 0.697–1.290, *p* < 0.001). The unadjusted model explained 12.2% of the variance (R^2^ = 0.122). Exploratory psychometric analyses provided preliminary support for use of the 33-item aggregate score. **Conclusions:** In this exploratory analysis of a non-probability sample, higher parental perceived eHealth literacy was associated with higher self-reported heat-protection practice scores. Given the cross-sectional design and reliance on self-reported measures, these findings should be interpreted as an association rather than evidence of a causal effect. The model explained a modest proportion of the variance. Whether changes in eHealth literacy are associated with subsequent changes in heat-protection practices should be examined in longitudinal and intervention studies.

## 1. Introduction

Extreme heat events have become an increasingly important public health concern as global temperatures continue to rise [1,2]. Children are particularly vulnerable to heat-related health effects because of physiological, developmental, and behavioral factors that may limit their ability to recognize heat stress, regulate their activity, maintain adequate hydration, and independently implement protective measures [3,4,5]. Exposure to high ambient temperatures has been associated with heat exhaustion, heatstroke, dehydration, electrolyte imbalance, respiratory exacerbations, emergency department visits, and other adverse pediatric health outcomes [3,6,7,8]. The increasing frequency and intensity of extreme heat events therefore require preventive strategies that address the specific needs of children.

Children’s ability to avoid heat-related harm depends substantially on the knowledge, decisions, and actions of their parents or caregivers. Recommended parental practices include maintaining adequate hydration, limiting strenuous activity and outdoor exposure during the hottest hours, providing access to cool environments, selecting appropriate clothing, monitoring weather warnings, recognizing symptoms of heat-related illness, and preparing an emergency response plan [9,10,11]. These practices may differ according to a child’s developmental stage, health status, and level of dependence on caregivers. Parents consequently require timely, understandable, and reliable health information to make appropriate decisions during periods of extreme heat.

Digital platforms have become an important source of health information for parents. However, access to online information does not necessarily ensure that the information can be located, understood, critically evaluated, and translated into appropriate action. eHealth literacy refers to the perceived ability to seek, find, understand, appraise, and apply health information obtained from electronic sources [12,13,14]. From an eHealth literacy perspective, parents’ perceived ability to access, understand, appraise, and apply digital health information may be associated with how they respond to extreme heat. Parents commonly use online health information when making decisions about their children’s health, although identifying reliable and appropriate information may remain challenging [15]. In the context of heat, warning messages that include actionable recommendations may improve understanding and promote protective responses [16]. More broadly, eHealth literacy has been positively associated with health-related behaviors, suggesting that it may also be associated with how digital health information is translated into practical protective action [17]. Previous studies have examined parental eHealth literacy in relation to online pediatric information seeking, chronic disease management, preventive behaviors, and caregiver outcomes [13,18,19,20,21]. Research has also shown associations between parental health literacy and child-related health outcomes, as well as between eHealth literacy and health-promoting lifestyles [22,23]. Nevertheless, most previous studies of parental eHealth literacy and online health information use have focused on disease-specific management, child health information seeking, or general parenting and health-related behaviors [24,25]. Although parental experiences and protective decisions during extreme heat have been explored [26], the relationship between parental eHealth literacy and self-reported practices for protecting children from extreme heat has not been clearly examined. This gap is particularly relevant because parents commonly seek child-related health information online and may require support in identifying, evaluating, and appropriately applying reliable digital health information [24,27]. Examining this association may help identify areas for future research and family-centered digital heat-health communication. This study aimed to examine the association between parental eHealth literacy and self-reported practices for protecting children from extreme heat in a sample of parents in Türkiye. From an eHealth literacy perspective, greater perceived ability to access, understand, appraise, and apply digital health information was expected to be associated with greater engagement in recommended heat-protection practices. Accordingly, we hypothesized that (H1) higher parental eHealth literacy would be associated with higher self-reported heat-protection practice scores and (H2) among parents with one child, this association would remain after adjustment for child age group and key parental sociodemographic characteristics. These hypotheses and the resulting findings were interpreted within an exploratory framework.

To our knowledge, this study is among the first to specifically examine the association between parental eHealth literacy and self-reported child heat-protection practices. By linking digital health information competencies with parental protective practices in the context of extreme heat, the study contributes to eHealth literacy research beyond disease-specific management and general health behaviors.

## 2. Materials and Methods

### 2.1. Study Design

A cross-sectional correlational study was conducted to examine the association between parental eHealth literacy and self-reported practices for protecting children from extreme heat. Data were collected between August and September 2023, during a period in which high ambient temperatures are commonly experienced in Türkiye. The study was reported in accordance with the Strengthening the Reporting of Observational Studies in Epidemiology (STROBE) guidelines for cross-sectional studies [28].

### 2.2. Participants and Sample Size

The study population comprised parents residing in Türkiye who had at least one child aged 0–18 years. The required sample size was calculated a priori using G*Power version 3.1.9.7 (Heinrich Heine University Düsseldorf, Düsseldorf, Germany). The analysis was specified under the F-test family using linear multiple regression: fixed model, R^2^ deviation from zero. With one predictor, an effect size of f^2^ = 0.03, α = 0.05, and 80% statistical power, the minimum required sample size was 264 participants, with an actual power of 0.801. An f^2^ of 0.03 was selected as a conservative small-effect assumption because it is close to Cohen’s conventional small-effect benchmark of f^2^ = 0.02 and substantially below the medium-effect benchmark of f^2^ = 0.15 [29,30]. This approach was also consistent with recommendations that sample-size calculations should be based on the smallest effect considered theoretically or practically meaningful for the research question [31]. To allow for potentially incomplete or unusable responses, 26 participants were added to the minimum required sample size (264 + 26 = 290), corresponding to an increase of 9.85%, or approximately 10% [32]. Ultimately, 313 parents who met the eligibility criteria were included in the study.

Participants were recruited using convenience and snowball sampling. The inclusion criteria were (i) being aged 18 years or older, (ii) residing in Türkiye, (iii) having at least one child aged 0–18 years, and (iv) providing voluntary informed consent. All 313 completed questionnaires met the eligibility criteria and were included in the analysis.

### 2.3. Data Collection Instruments

Data were collected using three instruments: (i) a researcher-developed Sociodemographic and Heat-Impact Information Form, (ii) the researcher-developed Practices for Protecting Children from Extreme Heat Survey, and (iii) the Turkish version of the eHealth Literacy Scale (eHEALS).

#### 2.3.1. Sociodemographic and Heat-Impact Information Form

The researchers developed this form to collect information on participants’ age, gender, educational level, employment status, perceived income level, place of residence, and number of children. The form also included items assessing parents’ self-reported experiences related to extreme heat, including a parent-reported perceived increase in the frequency of illness among their children attributed to hot weather during the summer, increased household expenses associated with cooling or temporary relocation, and consideration of permanent relocation to a cooler region.

#### 2.3.2. Practices for Protecting Children from Extreme Heat Survey

Previous studies have developed and validated instruments addressing heat-related knowledge and protective behaviors. For example, validated questionnaires have been developed to assess heatwave-related knowledge, awareness, risk perception, attitudes, practices, and behaviors in general adult populations [33,34]. In the child-health literature, validated parent-reported measures have also been developed for specific sun-protection behaviors, including sunscreen use, protective clothing, shade seeking, and limiting time outdoors [35,36,37]. However, these instruments either target heatwave-related behaviors in general populations or focus primarily on ultraviolet exposure and sun-protection-related psychosocial constructs. They do not comprehensively assess the broader range of parental practices relevant to protecting children from extreme heat, including hydration, activity modification, environmental cooling, symptom recognition, weather monitoring, and emergency preparedness. Therefore, the researchers developed the 33-item Practices for Protecting Children from Extreme Heat Survey based on a review of the relevant literature and institutional guidance on protecting children during extreme heat events [3,4,38,39,40]. The items were organized into four content domains: hydration and nutrition, activity and rest, clothing and sun protection, and heat-related illness prevention and response. These domains were used for conceptual organization of the items and were not treated as psychometrically validated subscales. Each item was rated on a 5-point Likert scale ranging from 1 (strongly disagree) to 5 (strongly agree). Total scores ranged from 33 to 165, with higher scores indicating greater self-reported agreement with recommended heat-protection practices.

Content validity was evaluated by a panel of seven faculty members with expertise in pediatric nursing and public health nursing. The item-level content validity index values ranged from 0.86 to 1.00, and the scale-level content validity index was 0.96. Values of 0.80 or higher were considered acceptable for content validity [41]. Internal consistency was assessed using Cronbach’s α, with a coefficient of ≥0.70 considered acceptable [42]. In the present study, Cronbach’s α for the total survey was 0.931, indicating high internal consistency. Additional item-level and dimensionality analyses were conducted in the present sample to evaluate whether use of the 33-item aggregate score was supported; these analyses are described in the Statistical Analysis and Results sections.

#### 2.3.3. eHealth Literacy Scale

The eHealth Literacy Scale (eHEALS), originally developed by Norman and Skinner and culturally adapted and validated in Turkish by Gencer, was used to assess parents’ perceived ability to find, understand, evaluate, and use health information obtained from electronic sources [43,44]. The scale consists of eight scored items rated on a 5-point Likert scale ranging from 1 (strongly disagree) to 5 (strongly agree). Total scores range from 8 to 40, with higher scores indicating higher perceived eHealth literacy. In the present study, Cronbach’s α coefficient for the scale was 0.932, indicating high internal consistency.

### 2.4. Data Collection Process

Following ethical approval obtained on 2 August 2023, data collection commenced electronically via Google Forms on 3 August 2023 and continued through September 2023. Participants were recruited using convenience and snowball sampling. The online survey link was distributed through social media platforms and professional networks, and recipients were encouraged to share the survey with other potentially eligible parents. No quotas, stratification procedures, or targeted recruitment strategies were used to increase participation among specific demographic groups, including men, individuals with lower educational attainment, or those residing outside city centers. Accordingly, the sampling strategy was not intended to produce a nationally representative sample of parents in Türkiye.

Individuals who accessed the survey were first presented with an electronic informed consent form. Only those who confirmed that they had read the study information and voluntarily agreed to participate were permitted to proceed to the survey instruments. All survey items were mandatory; therefore, no incomplete questionnaires were submitted. All 313 completed questionnaires met the eligibility criteria and were included in the final analysis. Completion of the survey required approximately 10–15 min.

### 2.5. Ethical Considerations

The study was conducted in accordance with the principles of the Declaration of Helsinki. Ethical approval was obtained from the Burdur Mehmet Akif Ersoy University Non-Interventional Clinical Research Ethics Committee on 2 August 2023 (Decision No. GO 2023/422). Before participation, all parents were informed about the purpose of the study, the voluntary nature of participation, confidentiality, and their right to withdraw without penalty. Electronic informed consent was obtained before participants were granted access to the survey instruments. No personally identifiable information, including names or national identification numbers, was collected. The study data were stored on a password-protected digital drive accessible only to the research team.

### 2.6. Statistical Analysis

Statistical analyses were performed using IBM SPSS Statistics for Windows, version 29.0 (IBM Corp., Armonk, NY, USA). Continuous variables were summarized as means, standard deviations, and ranges, and categorical variables as frequencies and percentages. Skewness and kurtosis coefficients were examined for the total scores of the Practices for Protecting Children from Extreme Heat Survey and the eHealth Literacy Scale; values between −1 and +1 were considered indicative of approximately normal distributions [45]. Internal consistency was assessed using Cronbach’s α.

Because the researcher-developed 33-item survey total score served as the dependent variable in the primary analysis, additional item-level and dimensionality analyses were conducted to evaluate whether use of an aggregate score was supported by the present data. Corrected item–total correlations and Cronbach’s α if an item was deleted were examined. Sampling adequacy and factorability were assessed using the Kaiser–Meyer–Olkin measure and Bartlett’s test of sphericity. Because the items were rated on a five-point ordinal scale, dimensionality analyses were based on polychoric correlations. The number of factors was examined using Velicer’s minimum average partial (MAP) test, component-based parallel analysis, and exploratory factor analysis with principal axis factoring and Promax rotation. The strength of a general factor was further evaluated using a Schmid–Leiman transformation, omega total and omega hierarchical, explained common variance (ECV) interpreted together with the percentage of uncontaminated correlations (PUC), and general-factor loadings [46]. These analyses were conducted in R version 4.5.2 (R Foundation for Statistical Computing, Vienna, Austria). These analyses were exploratory and were not intended as a formal validation of the instrument.

A simple linear regression analysis was performed to examine the association between parental eHealth literacy and self-reported heat-protection practice scores. Linearity and homoscedasticity were assessed using scatterplots of standardized residuals against standardized predicted values, and the normality of residuals was evaluated using a normal Q–Q plot. Potentially influential observations were assessed using standardized residuals and Cook’s distance, and the Durbin–Watson statistic was used as an indicator of residual independence. Because the model included a single predictor, multicollinearity assessment was not applicable. Results were reported using the unstandardized coefficient (B), standard error (SE), standardized coefficient (β), 95% confidence interval (CI), R^2^, adjusted R^2^, F statistic, and *p*-value.

To examine the potential influence of child age, an additional analysis was restricted to parents with a single child (*n* = 202), because the practice items referred generally to “my child” and no index child was defined for parents with more than one child. The original child-age categories were collapsed into a younger group (0–<1, 1–<3, and 3–<6 years) and an older group (6–<12 and 12–18 years). A multivariable linear regression model was fitted with the heat-protection-practice score as the dependent variable and eHealth literacy, child age group, parental age, gender, educational level, income status, and place of residence as predictors. Model assumptions were evaluated using residual diagnostics, and variance inflation factors (VIFs) were examined to assess multicollinearity. All statistical tests were two-sided, and *p* < 0.05 was considered statistically significant.

Two sensitivity analyses were conducted to address the developmental applicability of selected survey items. Because several nutrition-related items may have limited applicability during infancy, particularly before or during the transition to complementary feeding, the model was first repeated after excluding parents of children aged under one year. Five items identified as developmentally dependent—restricting highly caffeinated beverages, explaining the health risks of extreme heat, teaching the signs and symptoms of heatstroke, teaching appropriate first aid for heatstroke, and teaching the child to monitor weather forecasts—were then removed, and the model was repeated using the resulting 28-item score. Four of these items require the child’s own comprehension or learning, while the item concerning highly caffeinated beverages has limited relevance in younger children. Both the 33-item and 28-item models were estimated in the same restricted subgroup to distinguish the effect of sample restriction from the effect of item removal. These sensitivity analyses were intended to assess the robustness of the observed association to selected developmental applicability concerns and were not intended to establish age-specific validity of the survey.

## 3. Results

### 3.1. Participant Characteristics and Perceived Effects of Extreme Heat

The mean age of the 313 participants was 34.33 ± 6.33 years, with an age range of 19–52 years. As shown in Table 1, most participants were female (85.3%), university graduates (90.4%), and employed (71.6%). Most lived in city centers (78.9%), and 64.5% had one child. The 313 participating parents reported a total of 440 children. Of these children, 13.9% were aged 0–<1 years, 24.5% were aged 1–<3 years, 24.5% were aged 3–<6 years, 26.1% were aged 6–<12 years, and 10.9% were aged 12–18 years.

Regarding self-reported experiences related to extreme heat, 25.6% of participants reported a perceived increase in the frequency of illness among their children attributed to hot weather during the summer, 49.8% reported increased household expenses associated with extreme heat, and 33.2% reported considering permanent relocation to a cooler region.

### 3.2. Health Information Sources

Participants could report more than one source of health information. As shown in Table 2, healthcare professionals were reported as a source of health information by 69.3% of participants, while 52.1% reported using official institutional websites. Social media platforms were reported by 26.8% of participants, and blogs or online forums by 23.6%. Relatives and friends (11.2%) and television (10.5%) were the least frequently reported sources.

### 3.3. Parental Heat-Protection Practices

As shown in Table 3, the highest mean item scores were reported for never leaving the child unattended in a vehicle (M = 4.81, SD = 0.45), avoiding outdoor activities during peak heat hours (M = 4.64, SD = 0.56), and ensuring that the child drank more water than usual (M = 4.56, SD = 0.57). The lowest mean scores were reported for avoiding long car journeys (M = 3.63, SD = 1.01), teaching the child to monitor weather forecasts (M = 3.68, SD = 1.01), and resting with the feet elevated if the child experienced heat exhaustion (M = 3.71, SD = 0.97).

### 3.4. Psychometric Properties of the Heat-Protection Survey

All 33 items showed corrected item–total correlations above 0.30 (range: 0.352–0.642), and deletion of any individual item did not improve the overall internal consistency (Table 3). Sampling adequacy was high (KMO = 0.914), and Bartlett’s test of sphericity was significant (χ^2^(528) = 5092.1, *p* < 0.001). Based on the polychoric correlation matrix, the first eigenvalue was 13.81 and the second was 2.63, yielding a first-to-second eigenvalue ratio of 5.26; the first factor accounted for 41.8% of the total variance. Velicer’s MAP test and component-based parallel analysis supported a three-factor solution, which accounted for 53.3% of the total variance; the extracted factors were moderately to strongly correlated (r = 0.60–0.69). Schmid–Leiman analysis showed general factor loadings ranging from 0.45 to 0.70 across all 33 items. Omega total was 0.966, omega hierarchical was 0.816, and explained common variance was 0.643 (PUC = 0.634). Taken together, these exploratory findings indicated a multidimensional structure with substantial shared variance and provided preliminary support for use of the 33-item aggregate score in the primary analyses of the present sample. These findings should not be interpreted as establishing strict unidimensionality or full psychometric validation of the instrument.

### 3.5. Association Between eHealth Literacy and Heat-Protection Practices

The mean total score for the Practices for Protecting Children from Extreme Heat Survey was 137.74 ± 14.92 (range: 78–165), and the mean eHealth Literacy Scale score was 33.15 ± 5.25 (range: 12–40). The heat-protection practice score showed skewness and kurtosis values of −0.306 and 0.446, respectively, while the corresponding values for the eHealth literacy score were −0.563 and 0.520. These values supported approximate normality of both total scores.

As shown in Table 4, the unadjusted simple linear regression model was statistically significant, F(1, 311) = 43.357, *p* < 0.001. Higher parental eHealth literacy scores were positively associated with higher self-reported heat-protection practice scores (*B* = 0.993, SE = 0.151, β = 0.350, 95% CI: 0.697–1.290, *p* < 0.001). The model explained 12.2% of the variance in heat-protection practice scores (R^2^ = 0.122; adjusted R^2^ = 0.120). In the simple regression model, standardized residuals ranged from −3.399 to 2.737. Although one standardized residual exceeded an absolute value of 3, the maximum Cook’s distance was 0.149, indicating that this observation did not exert substantial influence on the model. Visual inspection of the standardized residual and normal Q–Q plots indicated no substantial violations of linearity, homoscedasticity, or residual normality. The Durbin–Watson statistic was 1.655, indicating no evidence of substantial residual autocorrelation.

### 3.6. Association Between eHealth Literacy and Heat-Protection Practices Among Parents with One Child

Given that the heat-protection practice items referred generally to “my child” and no specific index child was identified for parents with more than one child, child-age analyses were restricted to parents with only one child (*n* = 202). In this subgroup, the original child-age categories were collapsed into a younger-child group (0–<1, 1–<3, and 3–<6 years) and an older-child group (6–<12 and 12–18 years). Child age group alone was not associated with heat-protection practice scores (*B* = 0.347, SE = 2.438, β = 0.010, 95% CI: −4.461–5.155, *p* = 0.887).

Residual diagnostics indicated no substantial violations of the regression assumptions, and the VIF values indicated no problematic multicollinearity. In the multivariable model adjusted for child age group, parental age, gender, educational level, income status, and place of residence, parental eHealth literacy remained positively associated with heat-protection practice scores (*B* = 1.063, SE = 0.182, β = 0.386, 95% CI: 0.705–1.422, *p* < 0.001). Child age group was not independently associated with heat-protection practice scores (*B* = 0.194, SE = 2.514, β = 0.006, 95% CI: −4.764–5.152, *p* = 0.938). None of the other sociodemographic covariates were statistically significant. The overall adjusted model was statistically significant, F(8, 193) = 4.945, *p* < 0.001, explaining 17.0% of the variance in heat-protection practice scores (R^2^ = 0.170; adjusted R^2^ = 0.136; Table 5).

### 3.7. Developmental Sensitivity Analyses

Two sensitivity analyses were conducted to address the developmental applicability of selected survey items (Table 6). After excluding parents of children aged under one year (*n* = 158), the association between parental eHealth literacy and the full 33-item heat-protection practice score remained similar to that observed in the full single-child subgroup (B = 1.092, β = 0.394, *p* < 0.001 versus B = 1.063, β = 0.386, respectively). When the five developmentally dependent items were additionally removed, the association remained statistically significant and of similar magnitude (B = 0.921, β = 0.405, *p* < 0.001). The proportion of explained variance was 0.174 for the 33-item model and 0.196 for the 28-item model. Child age group was not a significant predictor in either sensitivity model.

## 4. Discussion

This study examined the association between parental eHealth literacy and self-reported practices for protecting children from extreme heat. Higher parental eHealth literacy scores were positively associated with higher heat-protection practice scores, and the unadjusted model explained 12.2% of the variance. This finding indicates an association between parental perceived eHealth literacy and self-reported heat-protection practices in this sample. However, the modest explained variance indicates that these practices are also likely to be influenced by sociodemographic, environmental, structural, and child-related factors not included in the model. Given the cross-sectional design, the observed association should not be interpreted as evidence that higher eHealth literacy causes better protective practices.

### 4.1. Interpretation of the Main Findings

The mean eHealth Literacy Scale score was 33.15 ± 5.25 on a possible range of 8–40, indicating generally favorable self-perceptions regarding the use of electronic health information. This score should not, however, be classified categorically as “high” because established cutoff values for eHEALS are lacking. The predominance of university graduates in the sample may have contributed to the relatively elevated mean score, as previous research has reported associations between educational attainment and digital or eHealth literacy [47,48]. The predominance of women, university graduates, and participants residing in city centers should also be considered when interpreting the findings, as the observed associations may not reflect those among parents with different educational, socioeconomic, geographic, or digital-access characteristics.

Healthcare professionals were the most frequently reported source of health information (69.3%), while 52.1% of participants reported using official institutional websites. The reported use of official institutional websites should be interpreted in the context of the online recruitment strategy, which may have preferentially reached parents with greater access to and familiarity with digital platforms. Therefore, the frequency of website use observed in this sample may not reflect information-seeking patterns among the broader parent population in Türkiye. In addition, because participants could select multiple sources, these findings do not identify a single primary or preferred source of health information. Previous research suggests that eHealth literacy may be associated with how individuals assess the credibility of health information sources [49]. However, the present study did not directly assess trust, source credibility appraisal, or the quality of the information accessed; therefore, these findings should not be interpreted as evidence of critically informed or evidence-based information-seeking behavior.

The positive association between eHealth literacy and heat-protection practice scores is broadly consistent with the literature reporting associations between eHealth literacy and parents’ and caregivers’ ability to locate, interpret, and use online health information in pediatric care and health-related decision making [18,19,20,21,23]. Individuals with limited health literacy may experience difficulty evaluating the quality and reliability of online health information, whereas stronger appraisal skills may facilitate more appropriate use of digital resources [50]. Nevertheless, the present study assessed perceived eHealth literacy rather than objectively tested digital information appraisal skills. The findings therefore indicate an association between two self-reported constructs rather than demonstrating that parents successfully identified or applied accurate online information.

Although the sample was relatively homogeneous with respect to several sociodemographic characteristics, substantial variability remained in the two continuous study measures: eHealth literacy scores ranged from 12 to 40, and heat-protection practice scores ranged from 78 to 165. This within-sample variability may have allowed the association between the two constructs to be detected despite the limited demographic diversity of the sample. Nevertheless, statistical significance should not be equated with large practical importance. The unadjusted model explained 12.2% of the variance in heat-protection practice scores, indicating that parental eHealth literacy represents only one component of a broader set of factors related to these practices. In the additional multivariable analysis restricted to parents with one child, parental eHealth literacy remained positively associated with heat-protection practice scores after adjustment for child age group, parental age, gender, educational level, income status, and place of residence. However, the adjusted model still explained only a modest proportion of the variance. Therefore, the findings should not be interpreted as suggesting that eHealth literacy alone determines parental heat-protection practices. Rather, eHealth literacy should be interpreted as one potential correlate within a broader set of factors related to parental heat-protection practices. Other relevant factors, including housing quality, access to air conditioning or other cooling resources, local climatic conditions, the child’s health status, and previous experience with heat-related illness, were not included in the model and may contribute to the remaining unexplained variance. Evidence indicates that socially and economically disadvantaged populations may experience greater heat vulnerability because of restricted access to adequate housing, cooling, healthcare, and adaptive resources [51].

The reliance on self-reported measures should also be considered when interpreting the observed association. Participants may have tended to report socially desirable heat-protection practices, particularly for behaviors that are widely perceived as responsible or expected parental actions. Because both eHealth literacy and heat-protection practices were assessed by self-report, shared method effects may also have contributed to the observed association. Consequently, the reported practice scores and the strength of the association may not fully reflect objectively observed parental behaviors. The direction and magnitude of this potential bias cannot be determined from the present data.

### 4.2. Patterns in Heat-Protection Practices

The item-level findings showed generally favorable self-reported responses to several immediate heat-protection practices. Never leaving a child unattended in a vehicle received the highest mean score. This finding is consistent with previous research examining caregiver attitudes and risk perceptions related to pediatric vehicular hyperthermia [52,53].

Relatively high scores were also observed for increasing children’s water intake, encouraging consumption of foods with a high water content, avoiding outdoor activities during the hottest hours, and maintaining a cool indoor environment. These practices are broadly consistent with recommended approaches to reducing children’s heat exposure and maintaining hydration during hot weather [54,55,56,57]. Hydration recommendations should nevertheless be adapted to the child’s age, developmental stage, activity level, feeding pattern, and health condition. Advice for infants, particularly exclusively breastfed infants, should not be assumed to be identical to recommendations for older children and adolescents.

In contrast, avoiding long car journeys, limiting strenuous physical activity, teaching children to monitor weather forecasts, and developing an emergency plan for heat-related incidents received comparatively lower scores. This pattern may suggest that participants reported greater familiarity with immediate protective actions than with anticipatory planning and activity modification. However, this interpretation should remain exploratory because the researcher-developed survey was not designed or psychometrically validated to distinguish between immediate and anticipatory dimensions.

The relatively lower score for limiting strenuous physical activity should not be interpreted as indicating that children should avoid physical activity generally. Heat risk during exercise reflects an interaction among ambient temperature, exercise intensity and duration, hydration status, clothing, acclimatization, and the child’s age and health status [58]. Guidance should therefore emphasize modifying the timing, intensity, and duration of strenuous activity during extreme heat; providing shaded rest periods; and ensuring developmentally appropriate hydration.

Weather monitoring and emergency preparedness also require developmentally appropriate approaches. Parents or caregivers remain primarily responsible for monitoring heat alerts and organizing protective actions for infants and young children. School-age children and adolescents may gradually be involved in recognizing heat warnings, modifying activities, identifying early symptoms, and seeking adult assistance. Previous literature has highlighted the value of early education in strengthening awareness of heat-related risks and supporting protective behaviors [54]. Family emergency planning may include identifying access to a cool environment, recognizing warning signs, initiating appropriate first-response measures, and knowing when urgent medical assessment is required.

### 4.3. Implications for Pediatric Nursing and Public Health

The following considerations reflect existing recommendations for child heat-health education and should not be interpreted as causal implications of the present findings.

Existing guidance supports the inclusion of heat-health education within pediatric nursing, family-centered care, and community health practice. Nurses and other healthcare professionals who interact directly with parents are well positioned to provide developmentally appropriate guidance on hydration, activity modification, environmental cooling, symptom recognition, weather monitoring, and emergency preparedness [59]. Education should distinguish between recommendations for infants, younger children, school-age children, adolescents, and children with chronic health conditions.

Parental education should also address the digital information skills required to locate and evaluate reliable heat-health guidance. Parental perceived eHealth literacy may be associated with confidence in finding, evaluating, and using online health information, but access alone does not guarantee that information is accurate, understandable, or appropriately applied. Health professionals should therefore direct families toward authoritative sources and teach practical criteria for evaluating online health information, rather than assuming that digitally active parents can independently distinguish credible information from misinformation [49,50].

Heat-health information may be delivered through face-to-face counseling, primary healthcare services, schools, digital platforms, social media, and community outreach. Community-based heat adaptation and education interventions have shown potential to improve heat-related knowledge, protective behavior, and selected health outcomes [51,60]. Nevertheless, digital communication should complement rather than replace direct professional guidance, particularly for families with limited digital access, lower literacy, inadequate housing, or restricted access to cooling resources.

Regional and national health authorities may also adapt international child-focused heat-health recommendations to local climatic, cultural, and service contexts. UNICEF guidance highlights the specific effects of heatwaves on children and the importance of practical caregiver action [61]. Locally adapted messages should provide clear, age-specific recommendations and should be accessible to families with differing educational, socioeconomic, and digital literacy levels. Given the increasing frequency and intensity of extreme heat events, integrating child-specific heat-health communication into public health preparedness is increasingly important [1].

## 5. Strengths and Limitations

A strength of this study was that data were collected during August and September, when high ambient temperatures are commonly experienced in Türkiye. This timing may have increased the salience of heat-related experiences and protective practices. The study also addressed an underexplored association between parental eHealth literacy and practices for protecting children from extreme heat.

Several limitations should be considered. The cross-sectional design precludes causal inference and does not establish the temporal direction of the observed association. The use of online convenience and snowball sampling may have introduced substantial selection bias by preferentially reaching parents with access to and familiarity with digital platforms. No quotas, stratification procedures, or targeted recruitment strategies were used to increase participation among men, parents with lower educational attainment, or those residing outside city centers. Consequently, the sample should not be considered representative of the overall parent population in Türkiye, and the findings should not be generalized to all parents in the country.

All variables were self-reported and may therefore have been affected by recall and social desirability bias. This concern is particularly relevant for socially sensitive practices, such as never leaving a child unattended in a vehicle. Moreover, eHEALS assesses perceived rather than objectively demonstrated eHealth literacy.

The researcher-developed heat-protection survey underwent exploratory item-level and dimensionality analyses in the present sample, which provided preliminary support for use of the 33-item aggregate score in the primary analyses. However, these findings do not constitute full psychometric validation of the instrument. The factor structure has not been independently confirmed, and test–retest reliability, responsiveness, criterion-related validity against observed behavior, and replication in an independent sample remain to be established. Several items also showed substantial ceiling effects, which may limit discrimination among participants reporting high levels of protective practices. Independent validation of the instrument in a separate and more heterogeneous sample is therefore warranted. In addition, some child-directed survey items may vary in applicability according to the child’s developmental stage, and age-specific applicability was not predefined in the original survey design. Sensitivity analyses indicated that the observed association remained similar after excluding parents of children aged under one year and after removing the five items identified as developmentally dependent.

Although an additional multivariable analysis was conducted among parents with one child, potentially relevant environmental and child-related factors, such as housing conditions, access to cooling resources, local climatic conditions, and child health status, were not assessed. In addition, although an age-related analysis was conducted in this subgroup, the broad pediatric age range and the need to collapse child ages into two groups limited detailed comparisons across specific developmental stages. Future studies should validate the survey in more diverse samples, examine narrower pediatric age groups using developmentally tailored heat-protection measures, and use longitudinal and intervention designs to clarify the robustness, direction, and practical implications of the observed association.

## 6. Conclusions

This study identified a positive association between parental perceived eHealth literacy and self-reported child heat-protection practices in this non-probability sample. Because both variables were self-reported and measured at a single time point, the findings should be interpreted as an association rather than as evidence of a causal effect.

Participants reported higher agreement with immediate protective practices, particularly avoiding leaving children unattended in vehicles, limiting outdoor exposure during the hottest hours, maintaining hydration, and supporting access to cooler environments. Comparatively lower agreement was observed for anticipatory practices, including weather monitoring, activity planning, and emergency preparedness. This descriptive pattern may be relevant for future research and educational planning, although the present design cannot determine which practices should be prioritized.

Whether changes in eHealth literacy are associated with subsequent changes in heat-protection practices should be examined in longitudinal and intervention studies conducted in more representative samples, using measures that extend beyond parental self-report.

## Figures and Tables

**Table 1:** Participant characteristics and heat-related experiences (*n* = 313).

Variables	Mean ± Standard Deviation	Min–Max	
Age (years)	34.33 ± 6.33	19.00–52.00	
		***n***	**%**
**Gender**	Female	267	85.3
	Male	46	14.7
**Education Level**	High School	30	9.6
	University	283	90.4
**Employment Status**	Employed	224	71.6
	Unemployed	89	28.4
**Income Status**	Income lower than expenses	86	27.5
	Income equal to expenses	148	47.3
	Income higher than expenses	79	25.2
**Place of Residence**	District	66	21.1
	City center	247	78.9
**Number of Children**	1	202	64.5
	2	95	30.4
	3 or more	16	5.1
Children’s Age Groups (*n* = 440) *	0–<1 years	61	13.9
	1–<3 years	108	24.5
	3–<6 years	108	24.5
	6–<12 years	115	26.1
	12–18 years	48	10.9
Impact of Extreme Heat on Children and Family **	Perceived increase in child illness due to hot weather	80	25.6
	Increase in household expenses (e.g., cooling-related energy costs)	156	49.8
	Considering permanent relocation due to heat	104	33.2

Note: * Child-age percentages were calculated based on the total number of children reported by the 313 participating parents (*n* = 440). ** Participants could select more than one response; therefore, percentages exceed 100%.

**Table 2:** Health information sources reported by participants (*n* = 313).

Information Sources	*n*	% *
Healthcare professionals	217	69.3
Official institutional websites	163	52.1
Social media platforms	84	26.8
Blogs and forum websites	74	23.6
Relatives and friends	35	11.2
Television	33	10.5

**Note:** *n*, Number of participants. * Participants could select more than one response; therefore, percentages exceed 100%.

**Table 3:** Heat-protection practice item scores and item-level psychometric properties (*n* = 313).

Survey Items	M	SD	r (i–t)	α If Deleted
Ensuring the child drinks more water than usual	4.56	0.57	0.449	0.930
Encouraging consumption of high-water-content foods (e.g., fruits, vegetables, yogurt)	4.48	0.58	0.524	0.929
Restricting the intake of highly caffeinated beverages (e.g., coffee, cola, energy drinks)	4.32	0.83	0.541	0.929
Providing light meals (e.g., vegetable-based dishes, salads)	4.05	0.78	0.535	0.929
Restricting high-calorie, sugar-sweetened beverages (e.g., iced tea and sodas)	4.03	0.95	0.523	0.929
Restricting excessively salty foods	4.01	0.83	0.596	0.928
Restricting excessively fatty foods	3.98	0.87	0.591	0.928
Restricting sugary products and confectionery	3.93	0.92	0.588	0.928
Restricting high-calorie snacks and fast food (e.g., chips, pastries, burgers)	3.86	0.93	0.533	0.929
Never leaving the child unattended in a vehicle	4.81	0.45	0.352	0.931
Avoiding outdoor activities during peak heat hours	4.64	0.56	0.465	0.930
Taking the child outdoors after sunset when temperatures are lower	4.51	0.61	0.446	0.930
Maintaining a cool indoor environment for the child	4.51	0.61	0.501	0.930
Ensuring regular rest in the shade during outdoor activities (e.g., pool or beach)	4.39	0.62	0.503	0.929
Avoiding direct exposure to air conditioning or fans	4.16	0.87	0.399	0.931
Ensuring adequate rest periods	4.12	0.84	0.528	0.929
Limiting strenuous physical activities (e.g., running, cycling)	3.76	0.96	0.456	0.930
Avoiding long car journeys	3.63	1.01	0.434	0.931
Choosing breathable cotton clothing that prevents excessive sweating	4.48	0.65	0.611	0.928
Choosing loose-fitting clothing	4.46	0.63	0.586	0.929
Ensuring the child wears a hat in direct sunlight	4.41	0.73	0.621	0.928
Choosing light-colored clothing	4.37	0.73	0.555	0.929
Applying sunscreen before going outdoors	4.14	0.98	0.544	0.929
Ensuring the child wears sunglasses in direct sunlight	3.92	1.04	0.562	0.929
Seeking shade during prolonged outdoor exposure	4.41	0.62	0.543	0.929
Giving the child a lukewarm shower or soaking their feet in cool water	4.31	0.68	0.498	0.929
Preventing sudden temperature shocks (e.g., rapid entry into cold water)	4.17	0.81	0.494	0.929
Explaining the health risks associated with extreme heat	4.08	0.83	0.577	0.929
Teaching the signs and symptoms of heatstroke	4.01	0.86	0.555	0.929
Teaching appropriate first aid for heatstroke	3.96	0.92	0.571	0.929
Developing an emergency plan for heat-related incidents	3.88	0.94	0.642	0.928
Resting with feet elevated if the child experiences heat exhaustion	3.71	0.97	0.530	0.929
Teaching the child to monitor weather forecasts	3.68	1.01	0.547	0.929

**Note:** Items were rated on a 5-point Likert scale ranging from 1 (strongly disagree) to 5 (strongly agree), with higher scores indicating greater self-reported agreement with the corresponding heat-protection practice. M = mean; SD = standard deviation; r (i–t) = corrected item–total correlation; α if deleted = Cronbach’s alpha if the item is deleted. Overall Cronbach’s α was 0.931, and the mean inter-item correlation was 0.30 (Pearson) and 0.40 (polychoric).

**Table 4:** Simple linear regression of eHealth literacy and heat-protection practices (*n* = 313).

Predictor	B	SE	β	t	*p*	95% CI for B Lower–Upper
Constant	104.804	5.064	-	20.697	<0.001	94.841–114.768
eHealth Literacy	0.993	0.151	0.350	6.585	<0.001	0.697–1.290

**Model Summary:** R = 0.350; R^2^ = 0.122; Adjusted R^2^ = 0.120; F(1, 311) = 43.357; *p* < 0.001; Durbin-Watson = 1.655. **Note:** B, Unstandardized coefficient; SE, Standard Error; β, Standardized coefficient; *p*, Statistical significance; CI, Confidence Interval. **Dependent variable:** Practices for Protecting Children from Extreme Heat Survey total score.

**Table 5:** Multivariable linear regression analysis of heat-protection practice scores among parents with one child (*n* = 202).

Predictor	B	SE	β	t	*p*	95% CI for B Lower–Upper
Constant	112.969	9.304	-	12.142	<0.001	94.618–131.319
eHealth literacy	1.063	0.182	0.386	5.845	<0.001	0.705–1.422
Older-child group ^1^	0.194	2.514	0.006	0.077	0.938	−4.764–5.152
Parental age	−0.136	0.196	−0.051	−0.695	0.488	−0.523–0.250
Male gender ^2^	−3.377	3.040	−0.075	−1.111	0.268	−9.373–2.619
University education ^3^	−2.760	3.888	−0.047	−0.710	0.479	−10.429–4.909
Income equal to expenses ^4^	1.261	2.372	0.042	0.532	0.596	−3.417–5.939
Income higher than expenses ^4^	0.892	2.722	0.026	0.328	0.743	−4.477–6.261
City center residence ^5^	−3.450	2.467	−0.093	−1.399	0.163	−8.316–1.415

**Model summary:** R^2^ = 0.170; Adjusted R^2^ = 0.136; F(8, 193) = 4.945; *p* < 0.001; Durbin–Watson = 1.798. **Note:** B, unstandardized regression coefficient; SE, standard error; β, standardized regression coefficient; CI, confidence interval. **Dependent variable:** Practices for Protecting Children from Extreme Heat Survey total score. ^1^ Reference category: younger-child group (0–<1, 1–<3, and 3–<6 years); older-child group comprised 6–<12 and 12–18 years. ^2^ Reference category: female. ^3^ Reference category: high school. ^4^ Reference category: income lower than expenses. ^5^ Reference category: district.

**Table 6:** Sensitivity analyses for the association between parental eHealth literacy and heat-protection practice scores.

Model	*n*	Items	B	SE	β	95% CI for B	*p*
A. All parents with one child, full score	202	33	1.063	0.182	0.386	0.705–1.422	<0.001
B. Children aged ≥ 1 year, full score	158	33	1.092	0.208	0.394	0.682–1.503	<0.001
C. Children aged ≥ 1 year, reduced score	158	28	0.921	0.168	0.405	0.589–1.253	<0.001

**Model summary:** Coefficients are shown for parental eHealth literacy. All models were adjusted for child age group, parental age, gender, educational level, income status, and place of residence. Model A corresponds to the single-child subgroup analysis reported in Table 5. Models B and C excluded parents of children aged under one year. The reduced 28-item score in Model C excluded five items identified as developmentally dependent. Model fit was F(8, 149) = 3.925, *p* < 0.001, R^2^ = 0.174 for Model B and F(8, 149) = 4.553, *p* < 0.001, R^2^ = 0.196 for Model C. B = unstandardized coefficient; SE = standard error; β = standardized coefficient; CI = confidence interval.

## Data Availability

The data supporting the findings of this study are available from the corresponding author upon reasonable request. The data are not publicly available due to ethical and participant privacy considerations.
